# Note on Secondary Variolous Ophthalmia

**Published:** 1855-03

**Authors:** 


### MEDICAL EXAMINER.

NEW SERIES.—NO. CXXIII.—MARCH, 1855.

### ORIGINAL COMMUNICATIONS.

### *Note on Secondary Variolous Ophthalmia.* BY DR. LITTELL.

*Mr. Editor* :—In an article published in the September number of the Examiner, I incidentally expressed the opinion, that the opacity of the cornea in what is termed secondary variolous ophthalmia was owing, rather to the actual death of the part from inadequate nutrition, than, as is commonly supposed, to the formation in that membrane of a pustule analogous to those of the primary disease. The later occurrence and different appearance of the supposed pustule, is attempted to be accounted for by the difference of texture between the cornea and the skin; but it has, in fact, none of the characteristics of a pustule, possesses, so far as is known, no specific property, and more closely resembles, both in its aspect and progress, a slough detached through the ulcerative process. Its primary seat appears to be the conjunctival covering of the cornea, but the intensity of the subsequent reaction may be such as to implicate also the more deeply seated tissues, producing purulent infiltration of the cornea, effusion into the anterior chamber, iritis, &c., and occasioning greater or less impairment of vision.

The pathological view suggested, is chiefly important for its practical bearing; and therefore it is that I have recalled attention to the subject. Of course, where the existence of general debility, atony of the extreme vessels, an altered condition of the fluids, and defective assimilation might almost certainly be presumed from the gravity of the previous constitutional malady, no prudent practitioner would think of employing depletory or antiphlogistic remedies with the same freedom as under other circumstances; but the importance of the organ and the severity of the inflammation, occupying his attention to the exclusion of the general depravation, might, nevertheless, betray him into the use of means from which he would not reap all the advantage that could be obtained, or which, indeed, by thwarting the restorative efforts of nature, might be positively injurious.

Regarded, however, as a slough occurring in a tissue of low organization, on the periphery of the system, and subject, therefore, to the operation of known laws, the indications of treatment are sufficiently apparent; imparting at once confidence to the physician and certainty to practice.

The Ol. morrhuæ with the spirits of turpentine as an alterative, a generous diet, the sulphate of quinine, and morphium to procure rest at night, are the chief general remedies which I have found necessary. Mackenzie recommends the tartrate of antimony so as to vomit and purge freely, during the acute stage of the inflammation, and subsequently has recourse to tonics. Where the internal parts of the eye become involved, calomel in small doses should be given in conjunction with the quinine, or as part of a course more actively antiphlogistic. The local inflammation, within certain limits, is a salutary movement, instituted to separate, in the first instance, a portion of the economy, now dead and useless, and afterwards to repair the consequences of its destruction. Should it transcend the degree required for this purpose, and threaten to implicate other tissues also, a few leeches to the temple will suffice for its abatement, and the relief of the congestion; but usually, no other topical treatment is demanded, than the use of atropia or belladonna to preserve the dilatation of the iris, and the application to the ulcer remaining after the detachment of the slough, of a weak solution of the nitrate of silver. Should further stimulation be indicated, the wine of opium, diluted or otherwise, may be advantageously prescribed.

*February 15th*, 1855.

